# Study protocol: The cross-sectional Uppsala weight gain in pregnancy study (VIGA study)

**DOI:** 10.48101/ujms.v128.8832

**Published:** 2023-02-10

**Authors:** Theodora Kunovac Kallak, Alice Zancanaro, Katja Junus, Anna-Karin Wikström, Inger Sundström Poromaa, Susanne Lager

**Affiliations:** Department of Women’s and Children’s Health, Uppsala University, Uppsala, Sweden

**Keywords:** Placenta, adipose tissue, biomarkers, pregnancy outcome, maternal obesity, fetal growth, birth weight, diet, exercise, excessive pregnancy weight gain

## Abstract

**Background:**

More than two in five Swedish women are overweight or obese when becoming pregnant. Maternal overweight or obesity and excessive pregnancy weight gain are associated with several adverse pregnancy outcomes. The underlying mechanisms that link maternal adiposity, diet, exercise, pregnancy weight gain with pregnancy outcome are incompletely understood.

**Methods:**

We describe the design for a cross-sectional study of pregnant women at Uppsala University Hospital, Sweden. All participants delivered by elective cesarean section before the onset of labor. At inclusion, participants answered two questionnaires concerning their dietary and exercise habits. Fasting maternal blood samples (buffy coat, plasma, serum) were collected. During the cesarean section, biopsies of maternal subcutaneous and visceral adipose tissues were obtained. Placental tissue was collected after delivery. All biological samples were processed as soon as possible, frozen on dry ice, and stored at −70 °C. Pregnancy outcomes and supplementary maternal characteristics were collected from medical records.

**Results:**

In total, 143 women were included in the study. Of these women, 33.6% were primiparous, 46.2% had a pre-pregnancy body mass index (BMI) over 25 kg/m^2^, and 11.2% of the offspring were born large for gestational age (LGA). Complete collection, that is both questionnaires and all types of biological samples, was obtained from 81.1% of the participants.

**Conclusions:**

This study is expected to provide a resource for exploration of the associations between maternal weight, diet, exercise, pregnancy weight gain, and pregnancy outcome. Results from this study will be published in peer-reviewed, international scientific journals. This study was approved by the Regional Ethics Review Board in Uppsala (approval no 2014/353) and with an amendment by the Swedish Ethical Review Authority (approval no 2020-05844).

## Introduction

Worldwide, the proportion of overweight women is increasing, with many women being overweight or obese when becoming pregnant. In 2020, over 40% of Swedish women were considered overweight or obese at registration for antenatal care, as measured by body mass index (BMI) (1).

Obesity or being overweight before and during pregnancy increases the risk of a variety of complications in both mother and child. Obese women, on average, take longer to get pregnant (2) and are more likely to suffer from miscarriages or stillbirths. In addition, these women are more likely to develop gestational diabetes, preeclampsia, depression, and require delivery by emergency cesarean section (3, 4). Infants of obese mothers are at higher risk of developing several complications throughout their life. As such, they risk being born large for gestational age (LGA), having congenital anomalies (5), cardiovascular disease, stroke, asthma, type 2 diabetes (6), as well as developing certain types of cancers (7, 8). Furthermore, maternal obesity has an intergenerational effect: children of obese mothers are at a higher risk of becoming obese themselves (3, 6).

Like maternal obesity, excessive pregnancy weight gain is also associated with pregnancy complications. Excessive pregnancy weight gain has been linked with an increased risk of maternal development of gestational hypertension or preeclampsia. Women gaining excessive weight during pregnancy are also more likely to give birth to a LGA or macrosomic baby (9). It has also been shown that women with excessive pregnancy weight gain tend to retain more weight postpartum (10). There are guidelines for recommended rate and total weight gain during pregnancy from the United States (US) Institute of Medicine (11). These guidelines propose that underweight women (BMI < 18.5 kg/m^2^) should gain a total of 12.5–18 kg during pregnancy. The recommended weight gain is then progressively lower for women of normal weight (BMI 18.5–24.9 kg/m^2^; recommended weight gain 11.5–16 kg), overweight (BMI 25.0–29.9 kg/m^2^; recommended weight gain 7–11.5 kg), and obesity (BMI ≥ 30 kg/m^2^; recommended weight gain 5–9 kg). However, among women in Sweden (regardless of BMI category), the actual range of weight gain is broader than the recommended (12).

In addition to BMI and weight gain during pregnancy, maternal exercise habits may also influence pregnancy outcome. The Swedish healthcare system recommends 2.5 h of exercise a week for expectant mothers. The benefits of exercise for pregnant mothers are not clear. Some evidence suggests that exercise has a positive effect on the physical and the psychological well-being of expecting mothers. Mothers who exercise pre-pregnancy have a lower risk of developing preeclampsia and gestational diabetes (13). Women exercising during their pregnancy have a slightly lower risk of LGA or small for gestational age baby (SGA) (14), and a lower risk of developing prenatal depression (15). Dietary and exercise interventions have been shown to lower the risk of excessive pregnancy weight gain, but do not reduce the risk of preeclampsia or macrosomia (16).

Although adverse short- and long-term consequences of maternal obesity and excessive pregnancy weight gain for children are well-established, much remains to be understood regarding the underlying mechanisms. The overall hypothesis for this study was that there is an interplay between maternal adipose tissue and the placenta affecting pregnancy outcome. To test this hypothesis, the VIGA study (VIktuppgång under GrAviditet [in Swedish, ‘weight gain in pregnancy’]) was established to provide careful characterization of a cohort of pregnant women with extensive biological sample collection. The VIGA study has the potential to substantially contribute to an understanding of the mechanisms concerning diet, exercise, maternal weight, pregnancy weight gain, and how these affect pregnancy outcomes.

## Methods

### Aim, design and setting of the study

The aim of the VIGA study was to establish a pregnancy database and biobank at Uppsala University. Our study focuses on pregnancy weight gain and further facilitates research relating to maternal obesity in pregnancy. By combining biological samples, clinical data, and questionnaires, the VIGA study intends to explore physiological and endocrinal consequences of maternal weight gain and obesity during pregnancy.

**Figure 1:**
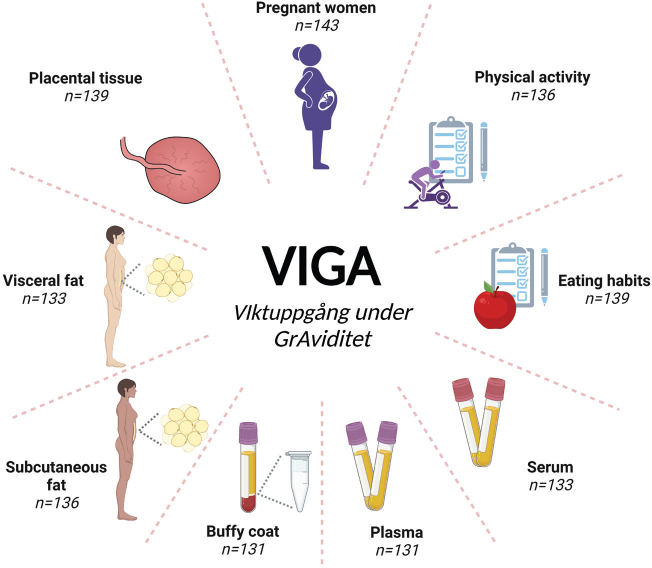
An overview of VIGA study. In the cross-sectional VIGA study, 143 women were included. For most of the women data from medical records, two questionnaires, as well as an extensive biological sample collection have been obtained.

The study design was cross-sectional, with two questionnaires and biological samples collected at time of delivery (Figure 1). Data on maternal and pregnancy characteristics were collected from medical charts. The study was conducted at Uppsala University Hospital in Uppsala, Sweden during 2014–2016. Before initiation of data collection, the study was approved by the Regional Ethics Review Board in Uppsala (approval no 2014/353) and later amended by the Swedish Ethical Review Authority (approval no 2020-05844).

### Characteristics of participants

Women undergoing elective cesarean section at Uppsala University Hospital were eligible for participation in the VIGA study. Additional participation requirements were: having a singleton pregnancy, being 18 years of age or older, no known blood-borne infections, as well as understanding Swedish (spoken and written). Before signing the consent form, the study was carefully described by the recruiting midwife/staff and the potential participant had an opportunity to ask questions.

### Data collection

Upon recruitment, participants answered two questionnaires. The first questionnaire consisted of 12 questions regarding eating habits. The second questionnaire focused on physical activity and consisted of 17 questions. Validated questionnaires were provided by the Swedish Food Agency (17). Minor changes to the questions were implemented to reflect diet and physical activity during pregnancy. Specifically, the first 10 questions of the dietary questionnaire focused on eating habits during the last 9 months instead of the last 12 months. Question 11 asked whether the respondent attempted changing her eating habits during pregnancy, rather than during the last 12 months. In the physical activity questionnaire questions 21 and 27 were changed to reflect activity during pregnancy, rather than the last year or present level of activity. The complete questionnaires (in Swedish) are available in Supplementary Table 1.

Medical records were reviewed, along with information collected throughout pregnancy in the maternal health care system and at the delivery ward. Sociodemographic information, obstetrical medical history, current medication, pregnancy complications, biochemical analyses, and neonatal information were also recorded.

### Biological sample collection

After inclusion, fasting maternal blood samples for buffy coat, plasma, and serum were collected. Serum samples were allowed to clot before being centrifuged and the serum was collected. Plasma samples were obtained in ethylenediamine tetraacetic acid -tubes. After centrifugation, buffy coat and plasma were separated and stored. During cesarean section, biopsies of maternal subcutaneous and visceral adipose tissue were taken. After delivery, a full thickness section from the central part of the placenta was obtained. Collected tissues were washed in sterile phosphate-buffered saline and then frozen immediately on dry ice. All samples were stored at −70 °C until further processing and analysis.

## Results

### Current findings

In total, 143 women were included in the VIGA study (Table 1). A majority of participants were born in Sweden and were cohabiting with a partner. Eighteen women (12.6%) conceived with the help of *in vitro* fertilization. A total of 46.2% of the women were overweight or obese when registered for maternal health care during early pregnancy. Fifty-eight women (40.6%) had an adequate weight gain according to the US Institute of Medicine recommendations based on their BMI category. Most of the remaining women (*n* = 62; 43.4%) gained more weight than recommended and had an excessive weight gain, while 19 women (13.3%) gained less weight and had an inadequate weight gain. Most of the women had a healthy pregnancy. Among recorded complications were five women with preeclampsia or hypertension and three women with diabetes. A large majority gave birth at term and approximately 11.2% of the children were born LGA, while only one was born SGA.

**Table 1:** Characteristics of VIGA study participants.

Variable	VIGA study (*n* = 143)
Maternal age, years	33.7 (5.2)
Primiparous	48 (33.6%)
Born in Sweden	122 (85.3%)
University education	94 (65.7%)
*Missing*	5 (3.5%)
Cohabiting with a partner	131 (91.6%)
Pre-pregnancy BMI, kg/m^2^	24.7 (17.3 – 44.1)
*Missing*	4
BMI ≥ 25 kg/m^2^	66 (46.2%)
*In vitro* fertilization	18 (12.6%)
Weight gain during pregnancy, kg	13.7 (4.3)
Inadequate weight gain*	19 (13.3%)
Adequate weight gain*	58 (40.6%)
Excessive weight gain*	62 (43.4%)
*Missing*	4 (2.8%)
Preeclampsia	2 (1.4%)
Hypertension	3 (2.1%)
Diabetes	3 (2.1%)
Gestational age, weeks	38.9 (36.9 – 41.6)
Infant sex, female	70 (49.0%)
Infant birth weight, grams	3606 (527)
*Small for gestational age*	1 (0.70%)
*Large for gestational age*	16 (11.2%)
*Missing*	4 (2.8%)

Parametric distributed data is provided as mean (standard deviation) while non-parametric data is given as median (min–max). Frequencies are given as total number (percentages). BMI, body mass index.

* Inadequate (less than the recommended), adequate (within the recommended range), and excessive (more than the recommended) weight gain calculated based on pre-pregnancy BMI category and US Institute of Medicine recommendations (11).

A total of 139 women (97.2%) reported eating habits and 136 (95.1%) reported physical activity during pregnancy. Serum was collected from 133 (93.0%) women, but plasma and buffy coat from only 131 (91.6%) women. Placental biopsies were collected from 139 (97.2%) women, with visceral and subcutaneous adipose tissue biopsies of 133 (93.0%) and 136 (95.1%) during cesarean section. The complete collection, those answering both questionnaires and donating all six types of biological samples, was obtained from 81.8% of the participants.

Using data from the Swedish National Board of Health and Welfare (18), a comparison of participant characteristics in the VIGA study was done for all women in Uppsala county and all women in Sweden giving birth during the same time period (presented in Table 2). This comparison suggests that, in general, participants of the VIGA study were on average 3.4 years older than the average age of women giving birth in Uppsala or Sweden. The VIGA study participants were more often multiparous and gave birth to more high birth weight infants.

**Table 2:** Comparison of VIGA study participants with pregnant women in the general population of Uppsala county and Sweden during the same time period.

Variable	VIGA study 2014–2016 (*n* = 143)	Uppsala county ‡ 2014–2016 (*n* = 11,383)	Sweden ‡ 2014–2016 (*n* = 328,688)
Maternal age (mean)	33.7 years	30.3 years	30.3 years
Primiparous (%)	33.6%	42.9%	43.0%
Pre-pregnancy BMI (mean) *	25.3 kg/m^2^	24.9 kg/m^2^	24.9 kg/m^2^
*Underweight (BMI <18.5)*	2.8%	2.4%	2.7%
*Normal weight (BMI 18.5–25)*	49.0%	57.4%	58.3%
*Overweight* *(BMI 25–30)*	33.6%	26.0%	25.5%
*Obese (BMI ≥ 30)*	11.9%	14.3%	13.6%
Infant birth weight **			
< *2,500 g*	0.7%	5.7%	4.8%
> *4,500 g*	5.6%	3.7%	3.6%
*Small for gestational age*	0.7%	2.4%	2.6%
*Large for gestational age*	11.2%	4.4%	3.5%

* Missing BMI data from four VIGA study participants (2.8%).

** Missing birth weight data from four VIGA study participants (2.8%).

‡Information on Uppsala county and Sweden retrieved from the Swedish National Board of Health and Welfare (18).

BMI, body mass index.

### Ongoing studies

Several projects are ongoing or planned utilizing the database and biological samples collected in the VIGA study. These projects include determination of placental RNA levels using sequencing, exploring potential associations between RNA levels and maternal pre-pregnancy BMI, pregnancy weight gain or fetal growth. In another project, a mass spectrometry-based metabolomics analysis of placenta and maternal plasma is currently being performed. This analysis is expected to generate data on amino acids, anthocyanins, fatty acids, flavonoids, lipids, oligolignols, organic acids, phenols, sterols, and sugars. Maternal adipose tissue will be analyzed using the same approach. In addition, maternal plasma proteins related to metabolism have been measured by proteomics using proximity extension assays (19). This assay simultaneously measures 92 different proteins, focusing on cell adhesion, cell surface receptor signaling pathways, cellular metabolic processes, and regulation of phosphorylation. In an upcoming study, maternal plasma proteins related to inflammation will be assessed using the same proximity extension assays technology. Further studies will be conducted to more completely describe the associations between maternal adiposity, pregnancy weight gain, diet, exercise, and fetal growth.

## Discussion

There is a need to better understand the impact of pregnancy weight gain and overweight/obesity as it relates to pregnancy health and outcome, with short- and long-term consequences for mother and child. This is in order to provide adequate care for overweight or obese pregnant women. Our project aim was establishment of a database and biobank for excessive maternal weight gain and obesity during pregnancy. Excessive weight gain and obesity are associated with pregnancy complications, such as accelerated fetal growth (3, 9, 20, 21). Accelerated fetal growth is associated with complications during delivery (22, 23). In addition, accelerated fetal growth has longstanding implications for the future health of an individual (6–8). Therefore, understanding the underlying mechanisms of accelerated fetal growth, along with how excessive weight gain and obesity impacts pregnancy, may further lead to strategies for managing these conditions.

### Strengths and limitations

Our study has several strengths. The database contains an extensive characterization of participants. Maternal health information, pregnancy, and child outcomes have been obtained from medical records. Complementing the information from medical records, the participants filled out two validated questionnaires regarding their dietary and exercise habits. This information allows for additional observations concerning laboratory data associated with the biological samples. Such observations are rarely included in studies of biological samples focusing upon accelerated fetal growth or maternal obesity/weight gain. Another strength of the study is the extensive biological sample collection, including maternal adipose tissue. Finally, a notable study strength is that most of the data and biological samples have been collected from a majority of study participants.

A study limitation is the low number of participants due to selecting those delivering by planned cesarean section, possibly resulting in a recruitment bias. When compared with the general population giving birth in Sweden during the same time period, participants in VIGA were older, less primiparous, and also gave birth to a higher proportion of large infants. However, only including women delivering through planned cesarean section is also an important study strength. This is because previous reports found that exposure to labor may affect placental RNA levels and activity of cellular signaling pathways (24–27). Therefore, the biological samples collected may better reflect the *in utero* environment rather than labor effects. Although the study is limited by no of participants and the recruitment bias, we anticipate the study has a capacity to contribute to an increased understanding of maternal excessive weight gain and obesity effects upon pregnancy outcome. This is achieved through careful characterization, the extensive data, and biological samples available from the participants.

### Summary

In conclusion, the VIGA study biobank and database allows for valuable laboratory discoveries regarding maternal excessive weight gain and overweight/obesity, as well as accelerated fetal growth. It is hoped that such discoveries may ultimately be used for the improvement of care for pregnant women.
