# Supplementary material for: Study protocol: The cross-sectional Uppsala weight gain in pregnancy study (VIGA study)
Source: Ups J Med Sci. 2023 Feb 10;127:10.48101/ujms.v128.8832. doi: 10.48101/ujms.v128.8832 (PMC10088726; doi:10.48101/ujms.v128.8832)
Supplement: Supplementary file 1 [file UJMS-128-8832-s001.pdf]

Study protocol: The cross-sectional Uppsala weight gain in pregnancy study (VIGA study).
Supplementary table 1. Dietary and physical activity questionnaires.

Nedan följder ett antal frågor om vad du brukar äta. Ha de senaste 9 månaderna i tankarna när du svarar på frågorna (dvs hur du har ätit under din graviditet). Kryssa för det alternativ som för varje fråga stämmer bäst överens med hur du brukar äta.
**1 Vilken typ av matfett brukar du vanligtvis använda på smörgåsen?**<br>*Markera ett alternativ, det vanligaste!*<br><br>1 ☐ Smör<br>2 ☐ Bregott, 80% fett<br>3 ☐ Bregott Mellan, Runda Bords, 60% fett<br>4 ☐ Hushållsmargarin (margarin i folie), tex Milda, HushållsEve, Ädel, Blåvitt<br>5 ☐ Bordsmargarin 80% fett, tex Flora, Linnéa<br>6 ☐ Lättmargarin, tex Lätta, Becel, Lätt&Lagom, LättLätt, Gaio, 30-40% fett<br>7 ☐ Benecol, Becel ProAktiv<br>8 ☐ Använder ej matfett på smörgås<br>9 ☐ Vet ej
**2. Vilken typ av matfett brukar du/ni vanligtvis använda i matlagningen hemma?**<br>*Markera ett alternativ, det vanligaste!*<br><br>1 ☐ Smör<br>2 ☐ Bregott, 80% fett<br>3 ☐ Bregott Mellan, Runda Bords, 60% fett<br>4 ☐ Hushållsmargarin (margarin i folie), tex Milda, HushållsEve, Ädel, Blåvitt<br>5 ☐ Bordsmargarin 80% fett, tex Flora, Linnéa<br>6 ☐ Flytande margarin<br>7 ☐ Matolja<br>8 ☐ Använder ej matfett<br>9 ☐ Vet ej
**3. Vilken typ av mjölk brukar du vanligtvis dricka/använda**<br>*Markera ett alternativ, det vanligaste!*<br><br>1 ☐ Standardmjölk eller gammaldags mjölk, 3% fett eller mer<br>2 ☐ Mellanmjölk 1,5% fett<br>3 ☐ Lättmjölk 0,5% fett<br>4 ☐ Minimjölk 0,1% fett<br>5 ☐ Dricker/använder inte mjölk

4. Vilken typ av fil eller yoghurt brukar du vanligtvis äta?
Markera ett alternativ, det vanligaste!

1 ☐ Fil (med eller utan bär), yoghurt (med eller utan frukt), kefir, A-fil, långfil (ca 3 % fett)
2 ☐ Mellanfil, yoghurt (med eller utan frukt), Onaka, Yoggi, Gaio Dofilus (1,5 – 2% fett)
3 ☐ Lättfil, lättyoghurt (med eller utan frukt), Valio yoghurt (0,01 – 0,5% fett)
4 ☐ Äter inte fil m.m.

5. Hur många skivor/bitar av följande brödsorter äter du vanligtvis under en vanlig vecka?

Markera för varje brödsort hur många skivor eller bitar du äter per dag eller per vecka om du inte äter den sorten dagligen.
En halv småfranska, ett halvt pitabröd eller motsvarande = en skiva mjukt bröd

	Per dag	Skrivor/bitar *eller*	Per vecka
1 Hårt bröd, tex rågknäcke, husman, sport	I_____I		I_____I
2 Hårt bröd, tex vete, frukost, kanel	I_____I		I_____I
3 Mjukt grovt bröd, fullkornsbröd	I_____I		I_____I
4 Limpa, rågsiktsbröd	I_____I		I_____I
5 Mjukt vitt bröd, tunnbröd, pita bröd	I_____I		I_____I
6 Äter inte bröd	☐ Gå till fråga 7		

6. Hur mycket matfett brukar du vanligen bre på en smörgås?
Jämför mängden i en vanlig portionsförpackning à 10 g.
Markera ett alternativ, det vanligaste!

1 ☐ Cirka ½ portionsförpackning
2 ☐ Cirka 1 portionsförpackning
3 ☐ Cirka 1½ portionsförpackning
4 ☐ Cirka 2 portionsförpackningar eller mer
5 ☐ Använder inte matfett på smörgås

7. Hur ofta äter du grönsaker och frukt? Svara per dag, vecka *eller* månad.

Ha de senaste 9 månaderna i tankarna. Svara på alla alternativ, men sätt endast ett kryss i den rad som passar bäst för varje matvara.

Grönsaker, baljväxter och rotfrukter = exempelvis broccoli, tomat, gurka, paprika, sallat, bönor, linser, morot, rödbeta, selleri och palsternacka. Försök också ta med rätter där grönsak ingår som blandad sallad, grönsaksblandningar, grönsaksjuice, grönsakssoppa eller gryträtter där grönsaker ingår.

Frukt och bär = exempelvis ett äpple, en apelsin, en banan, en klase druvor, en tallrik jordgubbar, eller frukt och bär som ingår i kräm, kompott eller fruktsallad.

Ange inte de tillfällen då intaget klart understiger en portion (tex en gurkskiva på smörgåsen, persiljekvist, bär som dekoration på tårtor m.m)

	Gånger per månad				Gånger per vecka						Gånger per vecka			
	<1*	1	2	3	1	2	3	4	5	6	1	2	3	≥4
Grönsaker och rotfrukter, alla typer (färska, frysta, konserv, stuvade m.m)	☐	☐	☐	☐	☐	☐	☐	☐	☐	☐	☐	☐	☐	☐
Frukt och bär, alla typer (färska, frysta, konserv, juice m.m)	☐	☐	☐	☐	☐	☐	☐	☐	☐	☐	☐	☐	☐	☐

**Mer sällan än 1 gång per månad eller aldrig*

Study protocol: The cross-sectional Uppsala weight gain in pregnancy study (VIGA study).
Supplementary table 1. Dietary and physical activity questionnaires.

8. Nedan följer en lista med olika matvaror. Hur ofta brukar du äta dessa matvaror?
Svara per dag, vecka *eller* månad.

Ha de senaste 9 månaderna i tankarna. Svara på alla alternativ, men sätt endast ett kryss i den rad som passar bäst för varje matvara.

	Gånger per månad				Gånger per vecka						Gånger per vecka			
	<1*	1	2	3	1	2	3	4	5	6	1	2	3	≥4
Potatis (kokt, mos, bakad, gratäng)	☐	☐	☐	☐	☐	☐	☐	☐	☐	☐	☐	☐	☐	☐
Pommes frites, stekt potatis	☐	☐	☐	☐	☐	☐	☐	☐	☐	☐	☐	☐	☐	☐
Fisk, skaldjur som huvudrätt	☐	☐	☐	☐	☐	☐	☐	☐	☐	☐	☐	☐	☐	☐
Kött, färs, fågel som huvudrätt	☐	☐	☐	☐	☐	☐	☐	☐	☐	☐	☐	☐	☐	☐
Korv som huvudrätt	☐	☐	☐	☐	☐	☐	☐	☐	☐	☐	☐	☐	☐	☐
Choklad och godis	☐	☐	☐	☐	☐	☐	☐	☐	☐	☐	☐	☐	☐	☐
Bullar, kakor, kex, tårta m.m.	☐	☐	☐	☐	☐	☐	☐	☐	☐	☐	☐	☐	☐	☐
Chips, salta pinnar, jordnötter, snacks	☐	☐	☐	☐	☐	☐	☐	☐	☐	☐	☐	☐	☐	☐
Hamburgare, kebab, varm korv med bröd	☐	☐	☐	☐	☐	☐	☐	☐	☐	☐	☐	☐	☐	☐
Ost, fett 24 – 40%	☐	☐	☐	☐	☐	☐	☐	☐	☐	☐	☐	☐	☐	☐
Ost 5-17% fett (nyckelhålsmärkt)	☐	☐	☐	☐	☐	☐	☐	☐	☐	☐	☐	☐	☐	☐
Pizza, matpajer, färdiga smörgåsar	☐	☐	☐	☐	☐	☐	☐	☐	☐	☐	☐	☐	☐	☐
Vispgrädde, creme fraiche 34 %	☐	☐	☐	☐	☐	☐	☐	☐	☐	☐	☐	☐	☐	☐
Glass	☐	☐	☐	☐	☐	☐	☐	☐	☐	☐	☐	☐	☐	☐
Läsk, saft	☐	☐	☐	☐	☐	☐	☐	☐	☐	☐	☐	☐	☐	☐

**Mer sällan än 1 gång per månad eller aldrig*

Supplementary table 1. Dietary and physical activity questionnaires.

9. Vad brukar du äta på vardagarna? Beskriv hur du brukar äta en vanlig dag.

Kryssa för den typ av måltid som överensstämmer bäst för varje måltidstillfälle. Ange endast ett kryss i den rad som passar bäst för varje måltid.

Kom ihåg mellanmål och ev. annat smååtande samt drycker

Måltidstillfälle	Måltider av typen				
	**Huvudmåltid** Lagad mat (t.ex. kött och potatis, soppa med bröd, sallad med bröd, pizza med sallad, hamburgertallrik	**Lättare måltid** t.ex. gröt/flingor med mjölk/fil, smörgåsar, soppa, sallad, omelett, varm korv, enkel hamburgare (ev. med dryck)	**Småmål** t.ex. kex, bulle, kaka, frukt, godis, glass (ev. med dryck)	**Dryck enbart** t.ex. kaffe, te, läsk, juice, mjölk, öl, vin	**Inget**
Frukost, morgonmål	☐	☐	☐	☐	☐
Måltid/måltider på förmiddagen	☐	☐	☐	☐	☐
Mitt-på-dagen mål (lunch)	☐	☐	☐	☐	☐
Måltid/måltider på eftermiddagen	☐	☐	☐	☐	☐
Middag, kvällsmål	☐	☐	☐	☐	☐
Mål sent på kvällen eller natten	☐	☐	☐	☐	☐

Study protocol: The cross-sectional Uppsala weight gain in pregnancy study (VIGA study).
Supplementary table 1. Dietary and physical activity questionnaires.

10. Vilken typ av mat eller kost äter du vanligen?
Markera ett alternativ, det vanligaste!

1 ☐ Blandkost, dvs. äter det mesta
2 ☐ Enbart laktovegetarisk kost, dvs. äter inte kött, fisk eller ägg
3 ☐ Mest laktovegetarisk kost, men äter ibland fisk och ägg
4 ☐ Vegankost, dvs. äter inte kött, fisk, ägg, mjölk och mjölkprodukter
5 ☐ Glutenfri kost
6 ☐ Annan kost, beskriv: ____

11. Har du under din graviditet ändrat eller försökt ändra dina matvanor för att äta mer näringsriktigt/hälsosamt?
Markera ett alternativ.

1 ☐ Ja, har börjat äta mer näringsriktigt/hälsosamt
2 ☐ Ja, har försökt men misslyckats
3 ☐ Nej, men har funderat på det
4 ☐ Nej, aldrig
5 ☐ Nej, anser att jag redan äter näringsriktigt/hälsosamt
6 ☐ Har redan ändrat matvanorna sen tidigare

12. Vad anser du vara de största problemen för dig när det gäller att försöka äta näringsriktigt/hälsosamt?

Kryssa för de tre alternativ som du upplever som de största problemen för din egen del

1 ☐ Inga problem → Gå vidare till fråga 13

2 ☐ Oregelbunden arbetstid
3 ☐ Hälsosam mat är tråkig/mindre lockande
4 ☐ Kunskap om matlagning
5 ☐ Tidsbrist
6 ☐ Känner mig iakttagen av andra
7 ☐ Begränsat urval av hälsosam mat när jag äter ute
8 ☐ Ointresserad av matlagning
9 ☐ Familjens eller vännernas smak
10 ☐ För stor förändring jämfört med mina nuvarande matvanor
11 ☐ Hälsosammare alternativ finns inte i affären
12 ☐ Hälsosammare alternativ finns inte hemma
13 ☐ Avstå från mat jag tycker om
14 ☐ Främmande eller ovanlig mat
15 ☐ Priset (på hälsosam mat)
16 ☐ Kan inte tillräckligt om hälsosam mat
17 ☐ Blir inte tillräckligt mätt
18 ☐ ”Experterna” bara ändrar sig hela tiden
19 ☐ Tar för lång tid att laga
20 ☐ Förvaringsmöjligheter
21 ☐ Begränsade matlagningsmöjligheter
22 ☐ Jag vill inte ändra mina matvanor
23 ☐ Annat, nämligen:

____

____

____

Study protocol: The cross-sectional Uppsala weight gain in pregnancy study (VIGA study).
Supplementary table 1. Dietary and physical activity questionnaires.

Följande frågor gäller din fysiska aktivitet, dvs. hur mycket du rört på dig. Vi vet att det inte är så lätt med fysisk aktivitet i slutet av en graviditet, men tänk på all form av fysisk aktivitet som du utför. Det kan vara på ditt arbete, under din fritid, i hemmet och utomhus. Tänk även på hur du förflyttar dig från en plats till en annan.
Med **fysisk aktivitet** menas all typ av rörelse som ger ökad energiomsättning. Fysisk aktivitet omfattar all typ av muskelaktivitet t.ex. städning, trädgårdsarbete, fysisk belastning i arbetet, hobbyverksamheter som golf, svampplockning, motion och träning.

**13. Hur många gånger har du de senaste 7 dagarna utfört mycket ansträngande fysisk aktivitet som du upplevde som mycket arbetsam och fick dig att svettas och/eller andas mycket kraftigare än normalt?**<br>*Exempel på sådana aktiviteter är löpning, skidåkning, fotboll, innebandy och andra bollsporter, aerobics eller ansträngande motionsgymnastik, och cykling i högre tempo. Svara endast för de aktiviteter som pågick minst 10 minuter i sträck.*<br><br>☐ Ingen gång → Gå till fråga 15.<br><br>I_____I gånger de senaste 7 dagarna
**14. Hur mycket tid spenderade du i genomsnitt per tillfälle på mycket ansträngande fysisk aktivitet?**<br><br>I_____I timmar I_____I minuter<br><br>☐ Vet inte/osäker
**15. Hur många gånger har du de senaste 7 dagarna utfört måttligt ansträngande fysisk aktivitet som du upplevde som måttligt arbetsam och fick dig att bli varm och/eller andas något kraftigare än normalt?**<br>*Exempel på sådana aktiviteter är gräsklippning och annat trädgårdsarbete, tyngre hushållsarbete, promenad i rask takt, cykling, simning eller andra motionsaktiviteter i måttligt tempo. Svara endast för de aktiviteter som pågick minst 10 minuter i sträck.*<br><br>☐ Ingen gång → Gå till fråga 17.<br><br>I_____I gånger de senaste 7 dagarna
**16. Hur mycket tid spenderade du i genomsnitt per tillfälle på måttligt ansträngande fysisk aktivitet?**<br><br>I_____I timmar I_____I minuter

☐ Vet inte/osäker

17. Hur många gånger har du de senaste 7 dagarna gått eller promenerat i en takt som <u>inte</u> fick dig att svettas eller bli varm eller andas kraftigare än normalt?

Detta gäller då du gått/promenerat som en del av arbete, i hemmet, för att göra ärenden och på din fritid. Svara endast för de aktiviteter som pågick minst 10 minuter i sträck.

☐ Ingen gång → Gå till fråga 19.

I_____I gånger de senaste 7 dagarna

---

18. Hur mycket tid spenderade du i genomsnitt per tillfälle på att gå eller promenera?

a) På vardagar

I_____I timmar I_____I minuter

☐ Vet inte/osäker

b) På helgen

I_____I timmar I_____I minuter

☐ Vet inte/osäker

---

19. Hur lång tid har du per dag under de senaste 7 dagarna tillbringat sittande i samband med arbete, studier, i hemmet och på din fritid?

Försök att uppskatta hur många timmar i genomsnitt. Exempel på detta är tid vid skrivbordet, hemma hos vänner, att sitta och äta eller prata, och att sitta och se på film eller TV.

a) På vardagar

I_____I timmar I_____I minuter

☐ Vet inte/osäker

b) På helgen

I_____I timmar I_____I minuter

☐ Vet inte/osäker

20. Hur fysiskt aktiv har du varit under de senaste 7 dagarna enligt din egen uppfattning?

☐ Mycket mer aktiv än vanligt!
☐ Mer aktiv än vanligt
☐ Varken mer eller mindre aktiv än vanligt
☐ Mindre aktiv än vanligt
☐ Mycket mindre aktiv än vanligt

21. Hur mycket har du rört dig och ansträngt dig kroppsligt på fritiden under de senaste 9 månaderna, dvs. under din graviditet?

Om din aktivitet varierat, så försök att ta ett genomsnitt. Obs! Kryssa endast i ett alternativ!

☐ Stillasittande fritid

Du ägnar dig mest åt läsning, TV, bio eller annan stillasittande sysselsättning på fritiden. Du promenerar, cyklar eller rör dig på annat sätt mindre än 2 timmar i veckan.

☐ Måttlig motion på fritiden

Du promenerar, cyklar eller rör dig på annat sätt under minst 2 timmar i veckan oftast utan att svettas. I detta inräknas också promenad eller cykling till och från arbetet, övriga promenader, ordinärt trädgårdsarbete, fiske, bordtennis, bowling.

☐ Måttlig, regelbunden motion på fritiden

Du motionerar regelbundet 1-2 gånger per vecka minst 30 minuter per gång med löpning, simning, tennis, badminton eller annan aktivitet som gör att du svettas.

☐ Regelbunden motion och träning

Du ägnar dig åt t.ex. löpning, simning, tennis, badminton, motionsgymnastik eller liknande vid i genomsnitt minst 3 tillfällen per vecka. Vardera tillfället varar minst 30 minuter per gång.

Supplementary table 1. Dietary and physical activity questionnaires.

22a. Hur mycket rör och anstränger du dig kroppsligt i ditt arbetet?
Markera det alternativ som passar bäst in på dig!

☐ Arbetar inte → Gå vidare till fråga 24a

☐ Övervägande stillasittande arbete

Exempel på sådana arbeten är skrivbordsarbete och montering av lättare delar.

☐ Lätt, men något rörligt arbete

Du går ganska mycket, men bär inte och lyfter inte tunga föremål.

☐ Måttligt tungt arbete

Du går mycket och lyfter eller bär ganska mycket.

☐ Tungt kroppsarbete

Du lyfter tunga föremål och anstränger dig mycket kroppsligt

22b. Hur många timmar per vecka arbetar du i genomsnitt med den typ av arbete som du angett ovan?

☐ mindre än 15 timmar per vecka
☐ 16-30 timmar per vecka
☐ 31-40 timmar per vecka
☐ mer än 40 timmar per vecka

23. Erbjuder din arbetsgivare någon form av ersättning för motion eller träning?
Du kan ange flera alternativ!

☐ Nej, sådana möjligheter finns inte
☐ Jag har möjlighet att träna/motionera på arbetstid och jag utnyttjar detta
☐ Jag har möjlighet att träna/motionera på arbetstid men jag utnyttjar inte detta
☐ Min arbetsgivare subventionerar träningskort eller liknande och jag utnyttjar detta
☐ Min arbetsgivare subventionerar träningskort eller liknande men jag utnyttjar inte detta
☐ Nej, men jag har andra möjligheter till träning

Vad? ____

☐ Vet ej

24a. Hur många dagar per vecka brukar du gå i rask takt till och från ditt arbete eller dina studier?
Räkna även med om du bara går delar av vägen

☐ Arbetar hemma eller arbetar/studerar inte → Gå vidare till fråga 26
☐ Går inte till arbetet/studierna → Gå vidare till fråga 25a
☐ 1-2 dagar per vecka
☐ 3-4 dagar per vecka
☐ 5 dagar per vecka

24b. Hur lång tid tar det i genomsnitt att gå i rask takt till ditt arbete eller dina studier?

☐ mindre än 10 minuter
☐ 10-20 minuter
☐ 20-30 minuter
☐ 30-40 minuter
☐ mer än 40 minuter

25a. Hur många dagar per vecka brukar du cykla på väg till och från ditt arbete eller dina studier?
Räkna även med om du bara cyklar delar av vägen

☐ Cyklar inte till arbetet/studierna → Gå vidare till fråga 26
☐ 1-2 dagar per vecka
☐ 3-4 dagar per vecka
☐ 5 dagar per vecka

25b. Hur lång tid tar det i genomsnitt att cykla till ditt arbete eller dina studier?

☐ mindre än 10 minuter
☐ 20-30 minuter
☐ 30-40 minuter
☐ mer än 40 minuter

26. Om du motionerar regelbundet, vilken/vilka motionsformer eller aktiviteter håller du på med?

____

____

____

☐ jag motionerar inte regelbundet

27. Vilket av påståendena nedan beskriver bäst vad du anser om omfattningen av din fysiska aktivitet under din graviditet?
Markera endast ett svarsalternativ

Under graviditeten har jag varit

☐ inte särskilt fysiskt aktiv och jag har inte för avsikt att bli mer fysiskt aktiv under de närmaste 6 månaderna
☐ inte särskilt fysiskt aktiv, men jag har funderat på att öka min fysiska aktivitet under de närmaste 6 månaderna
☐ fysiskt aktiv, men jag har bara varit det under de 6 första månaderna
☐ fysiskt aktiv och jag har varit det under hela graviditeten
☐ fysiskt aktiv, men hade velat röra på mig ännu mera

28. Brukar du välja att gå i trappor där det finns fungerande hiss eller rulltrappa?

☐ Ja, nästan alltid
☐ Ja, ibland
☐ Nej, nästan aldrig
☐ Nej, kan inte gå i trappor

29. Finns det något grönområde (större park eller liknande) eller skogsområde inom 5-10 minuters gångavstånd från där du bor?

☐ Ja
☐ Nej
☐ Vet ej

TACK för att du tagit dig tid att svara!
